# A novel strategy for screening mutations in the voltage-gated sodium channel gene of *Aedes albopictus* based on multiplex PCR-mass spectrometry minisequencing technology

**DOI:** 10.1186/s40249-023-01122-y

**Published:** 2023-08-15

**Authors:** Qunzheng Mu, Xin Zhao, Fengfeng Li, Wenyu Li, Xinxin Zhou, Xinchang Lun, Yiguan Wang, Dongdong Hua, Qiyong Liu, Di Xiao, Fengxia Meng

**Affiliations:** 1 National Key Laboratory of Intelligent Tracking and Forecasting for Infectious Diseases, National Institute for Communicable Disease Control and Prevention, Chinese Center for Disease Control and Prevention, Beijing, 102206 People’s Republic of China; 2 Weifang No. 2 People’s Hospital, Weifang, 261000 Shandong People’s Republic of China; 3 Weifang Medical College, Weifang, 261000 Shandong People’s Republic of China; 4 Beijing Daxing District Center for Disease Control and Prevention, Beijing, 102600 Beijing People’s Republic of China

**Keywords:** *Aedes albopictus*, VGSC gene, Mutation, Single nucleotide polymorphisms, Multiplex polymerase chain reaction-mass spectrometry mini-sequencing

## Abstract

**Background:**

The current prevention and control strategy for *Aedes albopictus* heavily relies on comprehensive management, such as environmental management and chemical control. However, the wide application of pyrethroids has facilitated the development of insecticide resistance, primarily via mutations in the voltage-gated sodium channel (VGSC) gene. This study aims to develop a novel strategy for detecting mutations in the VGSC gene in *Ae. albopictus* using multiplex PCR-mass spectrometry (MPCR-MS) minisequencing technology.

**Methods:**

We established a new strategy for detecting mutations in the VGSC gene in *Ae. albopictus* using MPCR-MS minisequencing technology. MPCR amplification and mass probe extension (MPE) were first used, followed by single nucleotide polymorphism (SNP) typing mass spectrometry, which allows the simultaneous detection of multiple mutation sites of the VGSC gene in 96 samples of *Ae. albopictus*. A total of 70 wild-collected *Ae. albopictus* were used to evaluate the performance of the method by comparing it with other methods.

**Results:**

Three target sites (1016, 1532, 1534) in the VGSC gene can be detected simultaneously by double PCR amplification combined with matrix-assisted laser desorption ionization–time-of-flight mass spectrometry, achieving a detection limit of 20 fg/μl. We applied this method to 70 wild-collected *Ae. albopictus,* and the obtained genotypes were consistent with the routine sequencing results, suggesting the accuracy of our method.

**Conclusions:**

MPCR-MS minisequencing technology provides a sensitive and high-throughput approach to *Ae. albopictus* VGSC gene mutation screening. Compared with conventional sequencing, this method is economical and time-saving. It is of great value for insecticide resistance surveillance in areas with a high risk of vector-borne disease.

**Graphical Abstract:**

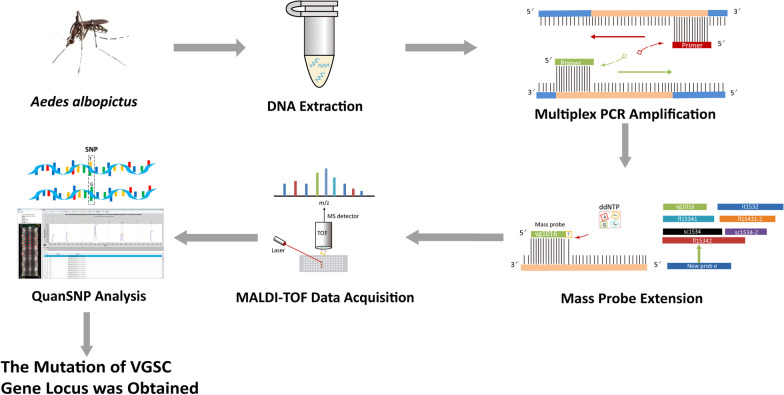

**Supplementary Information:**

The online version contains supplementary material available at 10.1186/s40249-023-01122-y.

## Background

*Aedes albopictus* is a major vector for the transmission of arboviruses, including dengue virus, chikungunya virus and Zika virus [1–3]. As one of the most invasive mosquito species with fast breeding and aggressive behaviour [4, 5], *Ae. albopictus* has successfully expended its presence across more than 70 countries worldwide [6]. Currently, *Ae. albopictus* is controlled by a combination of biological, chemical and physical methods, and chemical insecticides are primarily used to kill mosquitoes directly [7, 8]. Of these insecticides, pyrethroids are most widely used because of their broad spectrum, high efficiency and low toxicity to mammals [9, 10].

Voltage-gated sodium channels, encoded by the VGSC gene, are the main target of pyrethroid insecticides. Mutations in the VGSC gene could reduce the susceptibility of *Ae. albopictus* to pyrethroids, resulting in knockdown resistance [11]. The first sense mutation (F1534C) was discovered in Singapore in 2011 [12]. The sequence of VGSC gene loci in *Ae. albopictus* is determined based on the numbering of sodium channels in *Musca domestica* [13]. Multiple mutation sites have been found in the VGSC gene in *Ae. Albopictus* [14–20], and three of them (1016, 1532, and 1534) have been confirmed to be associated with knockdown resistance [11]*.* The mutation from GTA to GGA at codon site 1016 results in an amino acid change from valine (V) to glycine (G) [21]. At locus 1532, ATC (isoleucine, I) can be mutated to ATA (isoleucine, I) or ACC (threonine, T) [14]. At locus 1534, the wild-type TTC (phenylalanine, F) can be synonymously mutated to TTT (phenylalanine, F) or nonsynonymously mutated to TCC/TCG (serine, S), TGC (cysteine, C), TTG/CTG/CTC/TTA (leucine, L), CGC (arginine, R), or TGG (tryptophan, W) [12, 16, 18–20].

Currently, the detection of mutations in the VGSC gene in *Ae. albopictus* relies on DNA sequencing or allele-specific PCR (AS-PCR). DNA sequencing is most widely used and considered the gold standard for detecting VGSC mutations. However, DNA sequencing technology is not suitable for studies with a substantial number of samples due to its high cost and time requirements [19]. AS-PCR [19] has also been used to detect mutations in VGSC in *Ae. albopictus* but is only suitable for single-site detection, with relatively low specificity. Therefore, a rapid, accurate, economical, and multisite detection method for VGSC mutations in *Ae. albopictus* is an urgent need.

Matrix-assisted laser desorption ionization–time-of-flight mass spectrometry (MALDI–TOF MS) is often used to detect single nucleotide polymorphisms (SNPs). The main process of this method is to extract DNA from *Ae. albopictus* and amplify genes containing SNP targets by multiplex polymerase chain reaction (MPCR). Then, the mass probe is designed to extend the SNP sites detected. Finally, the mass-to-charge ratio (*m*/*z*) of the extended base identified by MALDI–TOF MS is used to determine the base type of the site. This method is also called MPCR-MS minisequencing technology [22]. At present, it is widely used in the clinic (e.g., microbiological testing and analysis) [23] and is very promising for SNP analysis because it can rapidly and simultaneously detect multiple targets [22, 24].

In this study, MPCR-MS minisequencing technology was used to detect 17 genotypes simultaneously at three VGSC gene mutation sites in *Ae. albopictus*. The established MPCR-MS minisequencing technology method was used to detect the VGSC gene in 70 samples of *Ae. albopictus* captured in the field, and the reliability of the method was evaluated.

## Methods

### Specimens and nucleic acids of *Ae. albopictus*

A total of 13 *Ae. albopictus* samples were used to establish the MPCR-MS minisequencing technology method. Samples of BJ1 were obtained from the National Institute for Infectious Disease Control and Prevention, Chinese Center for Disease Control and Prevention, in 2021. BJ1, the wild-type population susceptible to pyrethroids, served as control mosquitoes in this study (BJ1: V/V, I/I, F/F). The mutated-type or resistant mosquito populations were collected from seven cities (districts) of six provinces in China in 2020: YB2, MS32, LC1, LC7, MS5, YB8, HK33, LY6, SZ48, JN17, LY55, and YB6.

Another 70 wild-collected samples with unknown mutation status in the VGSC gene, sample numbers of HNLY1–HNLY30 and HNXC1–HNXC40, were used to test the performance of the newly established MPCR-MS minisequencing technology method. Sample collection information is provided in Table 1.

**Table 1:** *Aedes albopictus* sample collection information

Sample codes	Mutation status	Acquisition time	Collection method	Province/Municipality	City	District/County	Latitude and longitude
BJ1	No mutation/wild type/susceptible	October 2021	Artificial mosquito suction tube	Beijing	–	Changping	(116°30′E, 40°18′N)
YB2, YB6, YB8	Mutated-type/resistant	September 2020	Lamp trap	Chongqing	–	Yubei	(106°30′E, 29°35′N)
MS5, MS32	Mutated-type/resistant	July 2020	Lamp trap	Yunnan	Dehong	Mangshi	(98°59′E, 24°43′N)
LC1, LC7	Mutated-type/resistant	September 2020	Lamp trap	Yunnan	Puer	Lancang	(99°93′E, 22°55′N)
HK33	Mutated-type/resistant	September 2020	Lamp trap	Yunnan	Honghe	Hekou	(103°98′E, 22°52′N)
LY6, LY55	Mutated-type/resistant	July 2020	Lamp trap	Henan	Luoyang	Luolong	(112°46′E, 34°64′N)
SZ48	Mutated-type/resistant	May 2020	Lamp trap	Guangdong	Shenzhen	Luolong	(114°14′E, 22°57′N)
JN17	Mutated-type/resistant	July 2020	Lamp trap	Shandong	Jinan	Licheng	(117°03′E, 36°65′N)
HNLY1–HNLY30	Unknown	September 2022	Lamp trap	Henan	Luoyang	Luolong	(112°27′E, 34°40′N)
HNXC1–HNXC40	Unknown	September 2022	Lamp trap	Henan	Xuchang	Yuzhou	(113°26’E, 34°70′N)

– Means not applicable

DNA was extracted using a magnetic bead microtissue genomic DNA extraction kit (Bioteke Corporation, Wuxi, China). The three studied VGSC mutation sites (1016, 1532, 1534) were sequenced according to published papers [12, 21]. A total of 13 samples were used to establish the MPCR-MS minisequencing technology method for the detection of 14 mutation genotypes (V/G, G/G, I/T, T/T, F/S, S/S, F/C, C/C, F/L, L/L, S/C), as shown in Table 2. The 70 wild-collected samples were used for method validation, and routine sequencing was used to detect VGSC genes in the samples. An additional file shows this in more detail (see Additional file 1).

**Table 2:** Sites in the *Aedes albopictus* VGSC gene targeted by first-generation sequencing technology

Sample code	VGSC gene sequencing results		
	Locus 1016	Locus 1532	Locus 1534
BJ1	GTA/GTA	ATC/ATC	TTC/TTC
YB2	GTA/GGA	ATC/ATC	TTC/TGC
MS32	GGA/GGA	ATC/ATC	TTC/TTC
LC1	GTA/GTA	ATC/ACC	TTC/TCC
LC7	GTA/GTA	ACC/ACC	TTC/TTC
MS5	GTA/GTA	ATC/ATC	TCC/TCC
YB8	GTA/GTA	ATC/ATC	TGC/TGC
HK33	GTA/GTA	ATC/ATC	TTC/TTG
LY6	GTA/GTA	ATC/ATC	TTC/CTC
SZ48	GTA/GTA	ATC/ATC	CTC/CTC
JN17	GTA/GTA	ATC/ATC	TTC/TTA
LY55	GTA/GTA	ATC/ATC	TTA/TTA
YB6	GTA/GTA	ATC/ATC	TCC/TGC

### Design of PCR amplification primers and extension probe for VGSC gene variant sites

Primer Premier software (http://www.premierbiosoft.com/primerdesign/) was used for primer design. Two sets of PCR primers were designed to amplify the three VGSC target sites for multiplex PCR (Table 3). The lengths of the primers ranged from 18 to 22 bp, with a 10 bp fixed sequence (ACGTTGGATG) added to the 5' end of each primer.

**Table 3:** Primer sequences for PCR amplification of *Aedes albopictus* VGSC gene target sites

Target site	Primer sequences^a^	
	Forward primer sequence	Reverse primer sequence
1016	1016-F: acgttggatgCCACCGTAGTGATAGGAAATCT	1016-R: acgttggatgGTTGGCGATGTTGGACTTGA
1532/1534	3234-F acgttggatgGCGTACCTGTGTCTGTTC	3234-R: acgttggatgTTCTTCTTCTGCTCGTTGA

^a^The 10-bp fixed sequence added to the 5' end of the primer is indicated by lowercase letters

MALDI–TOF MS quality difference probes for mutation sites were designed by using IntelliBio genetic locus analysis software (V2.0, IntelliBio, Qingdao, China) (Table 4). The length of the mass probe was between 17 and 28 bp. The molecular weight was designed to be 4–9 kDa with a minimal difference of 16 Da between these probes. The probes vg1016, fl15341, sc1534 and fl15342 can be extended in one reaction tube (denoted as tube A), and probes it1532, fl15341-2 and sc1534-2 can be extended in another reaction tube (denoted as tube B).

**Table 4:** Primer sequences for *Aedes albopictus* VGSC gene MPE for target site detection

Target site	Mass probe name	Mass probe sequence (5'-3')	Mass probe mass (Da)	Extension base of nonvariant	Extended mass of MPE of nonvariant (Da)	Extension base of variant	Extended mass of MPE of variant (Da)	Extension base of variant	Extended mass of MPE of variant (Da)	Extension base of variant	Extended mass of MPE of variant (Da)
1016	vg1016	GGCTAAGAAAAGGTTAAGT	5924.8	T	6221.8	G	6197.8	–	–	–	–
1532	it1532	CTACTTCGTGTTCTTCA	5102.4	T	5444.4	C	5375.4	–	–	–	–
1534	fl15341	GTTGAGGGTGAAGAACGACCCGA	7162.6	T	7459.6	C	7475.6	–	–	–	–
	fl15341-2	TACTTCGTGTTCTTCATCATC	6313.2	T	6655.2	C	6586.2	–	–	–	–
	sc1534	TTGAGGGTGAAGAACGACCCG	6520.2	T	6817.2	C	6833.2	G	6793.2	–	–
	sc1534-2	TCTACTTCGTGTTCTTCATCATCT	7210.8	T	7552.8	C	7483.8	G	7523.8	–	–
	fl15342	AGGGTGAAGAACGACCC	5253.4	C	5566.4	G	5526.4	A	5595.4	T	5550.4

### Establishment of the MPCR-MS minisequencing technology method

**Fig. 1:**
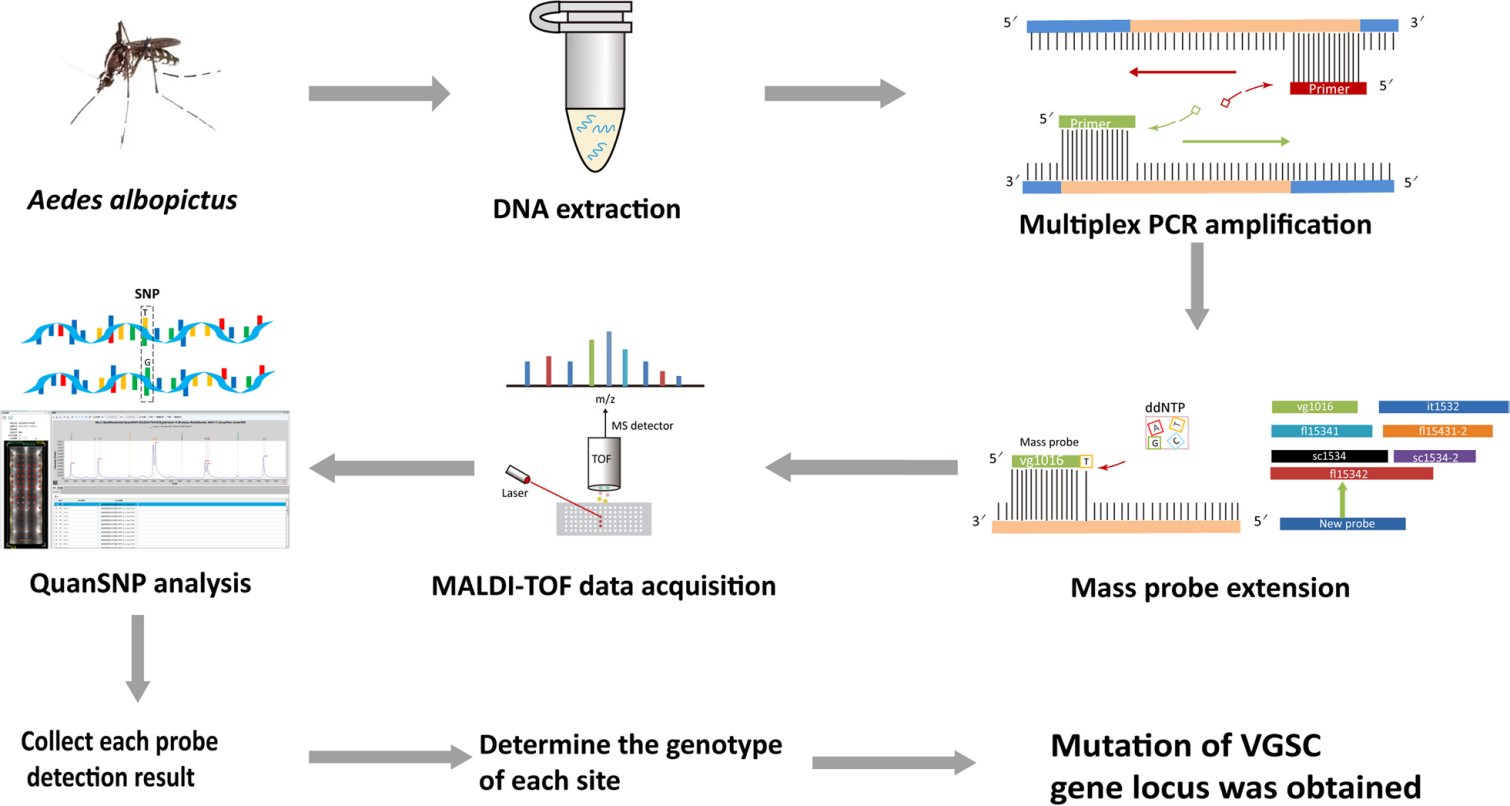
Workflow of MPCR-MS minisequencing technology for *Aedes albopictus* VGSC mutation detection. *MALDI–TOF* Matrix-assisted laser desorption ionization–time-of-flight, *SNP* Single nucleotide polymorphism, *VGSC* Voltage-gated sodium channel

In *Ae. albopictus,* the analysis method was developed by using the wild-type and mutated VGSC genes as templates. Whole buffer without template DNA served as the blank control (nucleic acid-free water was used instead of template DNA). The operation procedure mainly included DNA extraction, multiplex PCR amplification, mass probe extension (MPE), MALDI–TOF MS data acquisition and QuanSNP analysis. Each probe detection result was collected, the genotype at each site was determined, and then the mutation of the VGSC gene locus could be determined (Fig. 1).

DNA was extracted from the whole tissues of *Ae. albopictus* using a 96-well magnetic bead micro cell/tissue genome DNA extraction kit (Bio Teke, Wuxi, China) according to the manufacturer’s instructions. The extracted DNA was stored at − 20 °C.

The MPCR can simultaneously amplify two DNA fragments, with 1016 sites located in one DNA segment. The 1532 and 1534 sites were located in another DNA sequence. The MPCR instrument was the same as the ordinary sequencing PCR instrument. The MPCR was a 10 μl reaction system, including 2 × EasyTaqPCR Super Mix 5 μl, primers (1016-F, 1016-R, 3234-F, 3234-R) at 10 μmol/L each 0.5 μl, DNA template 2 μl, and ddH_2_O 1 μl. The reaction conditions were 94 °C for 5 min; 35 cycles of 94 °C for 30 s, 55 °C for 30 s, and 72 °C for 30 s; 72 °C for 8 min; and storage at 4 °C.

Shrimp alkaline phosphatase (SAP) digestion was conducted after multiple PCRs to eliminate free deoxygenated nucleoside triphosphates (dNTPs). The reaction system included 5 μl of PCR products and 2 μl of SAP mixture (1.53 μl of ddH_2_O, 0.17 μl of SAP buffer, 0.3 μl of SAP enzyme); the reaction conditions were 37 °C for 40 min, 85 °C for 5 min, and storage at 4 °C.

MPE was conducted by single-base extension of SAP products and mixed mass probes. The reaction system included 7 μl of SAP digestion products and 4 μl of mass probe mixture (1 μl of E-ddNTPmix, 1.4 μl of mass buffer, 0.6 μl of mass enzyme, 1 μl of mass probe). The reaction conditions were as follows: 95 °C for 30 s, followed by 52 °C for 5 s, 80 °C for 5 s, and 5 cycles, and then the entire process was repeated for 40 cycles before 72 °C for 3 min and finally stored at 4 °C.

An appropriate amount of resin was added to the MPE product for desalination and purification. First, 14 μl of deionized water was added to each reaction well, and then the resin was added, followed by centrifugation at 1006.2 × *g* for 1 min to prevent the resin from adhering to the tube wall. The sample plate was mixed with resin in a flip mixer at 10.1 × *g* for 30 min. After mixing, the sample was centrifuged at 1006.2 × *g* for 1 min, and the supernatant was tested.

Finally, MPE products were detected by MALDI–TOF MS. The OligoPlate target plate and matrix solution (3-hydroxypyridin-2-carboxylic acid: 3-HPA) were removed, the matrix solution was mixed thoroughly before use, preferably sonicated for 2 min, and a mixture of matrix and sample supernatant was prepared in a 1:1 volume ratio. 3-HPA (1 μl) was dropped at the centre of the sample target. Then, 1 μl of purified supernatant was mixed with 1 μl of 3-HPA. A 1 μl mixture was added onto the substrate target plate and allowed to dry. Then, the crystallized sample was tested using MALDI–TOF MS. The ratio of the mass of charged ions to their charge (*m*/*z*) was analysed to distinguish between different probes and probe extensions in the tested substance, and then the base at the extension position was determined. The detection results of the sites were exported as an Excel table.

Details can be found in our previous studies on detecting COVID-19 and *Mycoplasma pneumoniae* [22, 24].

### Determination of the detection limit

The DNA plasmid without mutations in the VGSC gene was synthesized and used to determine the lowest detection limit (LDL) of this method. The DNA plasmid was synthesized by Sangon Biotech (Shanghai, China), and the carrier was pUC57. The primers identified in this study were used for gene synthesis of the amplified sequences, including three mutation sites of the VGSC gene, and a total of 4 μg of dry powder plasmid was synthesized. The original concentration of nucleic acid was quantified and diluted along a six-concentration gradient (2 pg/μl, 200 fg/μl, 20 fg/μl, 2 fg/μl, 200 ag/μl, 20 ag/μl), and other reaction conditions remained unchanged.

### Application technology

The established MPCR-MS minisequencing technology method and conventional sequencing method were tested on 70 wild *Ae. albopictus* collected in Henan Province, China.

### Statistical analysis

Excel 2021 (Microsoft, Washington, USA) was used to conduct statistical analysis. Sample detection results were obtained from MALDI–TOF MS in Excel format (Intelligene Biosystems, IntelliBio, Qingdao, China), and then descriptive statistical analysis was performed.

## Results

### MPCR-MS mini-sequencing-based technology establishment and optimization

The three VGSC target sites (1016, 1532 and 1534) were amplified by double PCR. The original *m/z* peaks of the seven used mass probes (vg1016, it1532, fl15341, fl15341-2, sc1534, sc1534-2, fl15342) were 5924.8 ± 3.0, 5102.4 ± 3.0, 7162.6 ± 3.0, 6313.2 ± 3.0, 6520.2 ± 3.0, 7210.8 ± 3.0 and 5253.4 ± 3.0, respectively (mass error less than 500 mg/L, the same below; see Additional file 2a). The *m/z* peaks of the seven probes used in mass probe extension (MPE) were 6221.8 ± 3.0, 6197.8 ± 3.0, 5444.4 ± 3.0, 5375.4 ± 3.0, 7459.6 ± 3.0, 7475.6 ± 3.0, 6655.2 ± 3.0, 6586.2 ± 3.0, 6817.2 ± 3.0, 6833.2 ± 3.0, 6793.2 ± 3.0, 7552.8 ± 3.0, 7483.8 ± 3.0, 7523.8 ± 3.0, 5566.4 ± 3.0, 5526.4 ± 3.0, 5595.4 ± 3.0 and 5550.4 ± 3.0, respectively, and the extended bases were T, G, T, C, T, C, T, C, T, C, G, T, C, G, C, G, A and T (see Additional file 2b). For the blank control without DNA template, peaks in the mass probe extension are given in Additional file 2. c. An additional file shows this in more detail [see Additional file 2].

**Fig. 2:**
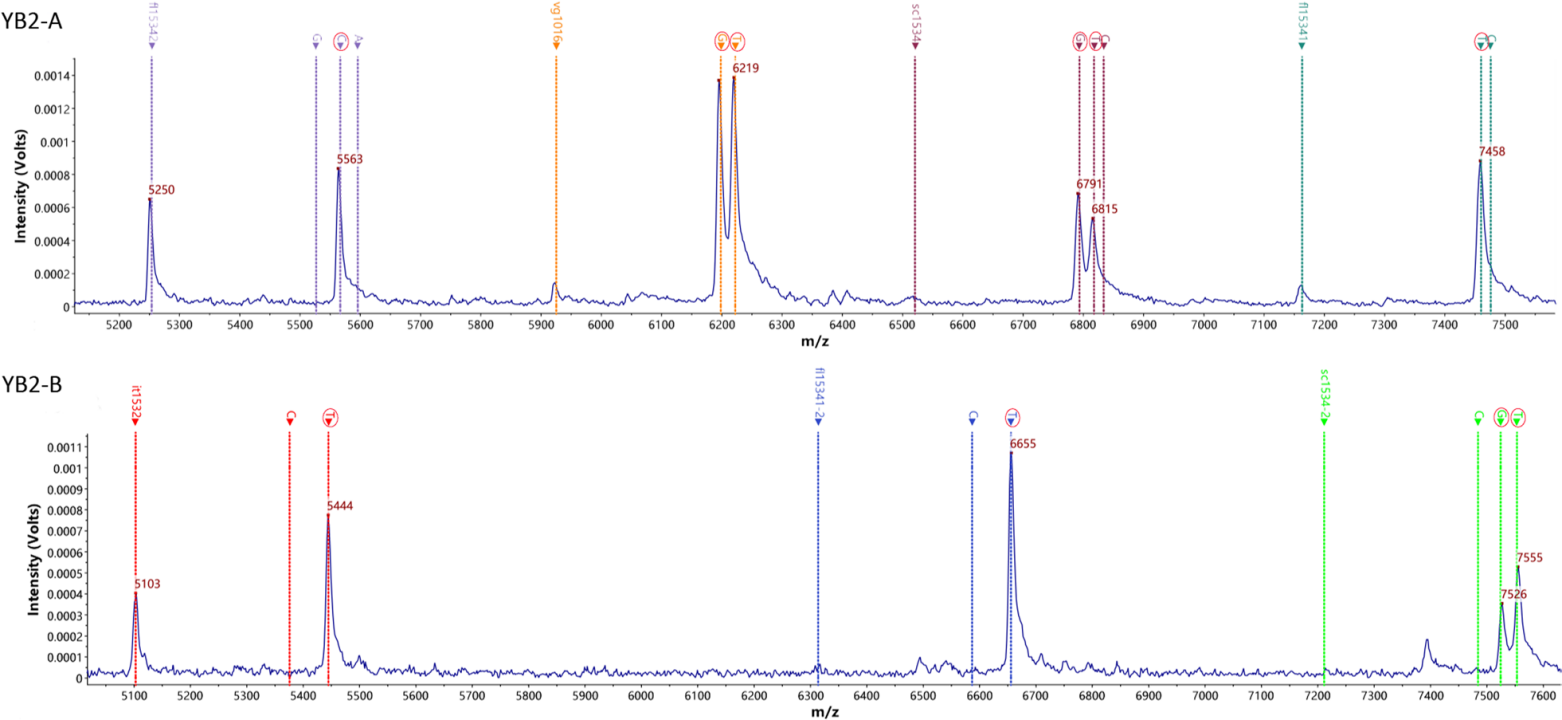
Sample (YB2) target site peaks of the mass probe extension. The detection result of the vg1016 probe is T/G. The detection result of the it1532 probe is T. The detection result of the fl15341/FL15341-2 probe is T. The detection result of the sc1534/SC1534-2 probe is T/G. The detection result of the fl15342 probe is C

For a better illustration of Additional file 2, a detailed explanation is given using sample YB2 as an example, as shown in Fig. 2. For site 1016, the wild-type homozygote genotype is GTA/GTA, where the second base T can be mutated into G. The vg1016 probe detection revealed two peaks, corresponding to T and G, respectively, suggesting wild-type/mutant heterozygote GTA/GGA. For site 1532, the wild-type homozygote genotype is ATC/ATC, where the second codon T can mutate into C. The it1532 probe detection result is T, suggesting a wild homozygote (ATC/ATC). For site 1534, the wild-type homozygote is TTC/TTC, and all three bases in this codon have been reported to be polymorphic. The detection result for the fl15341/fl15341-2 probe is T, for sc1534/sc1534-2 is T/G, and for the fl15342 probe is C, suggesting that this locus genotype is a wild-type/mutant heterozygote TTC/TGC genotype. The comprehensive detection results show the specific genotypes of three loci of the VGSC gene in the sample.

The VGSC gene loci of 13 *Ae. albopictus* mosquitoes were identified accurately using our MPCR-MS minisequencing technology method, which was confirmed using gene sequencing (Table 5).

**Table 5:** Detection results of target sites in the *Aedes albopictus* VGSC gene by MPCR-MS minisequencing technology

Sample code	MPCR-MS mini-sequencing detection results					Genotype			Comparison with gene sequencing
	vg1016	it1532	fl15341/ fl15341-2	sc1534/ sc1534-2	fl15342	Locus 1016	Locus 1532	Locus 1534	
BJ1	T	T	T	T	C	GTA/GTA	ATC/ATC	TTC/TTC	Consistent
YB2	T/G	T	T	T/G	C	GTA/GGA	ATC/ATC	TTC/TGC	Consistent
MS32	G	T	T	T	C	GGA/GGA	ATC/ATC	TTC/TTC	Consistent
LC1	T	T/C	T	T/C	C	GTA/GTA	ATC/ACC	TTC/TCC	Consistent
LC7	T	C	T	T	C	GTA/GTA	ACC/ACC	TTC/TTC	Consistent
MS5	T	T	T	C	C	GTA/GTA	ATC/ATC	TCC/TCC	Consistent
YB8	T	T	T	G	C	GTA/GTA	ATC/ATC	TGC/TGC	Consistent
HK33	T	T	T	T	C/G	GTA/GTA	ATC/ATC	TTC/TTG	Consistent
LY6	T	T	T/C	T	C	GTA/GTA	ATC/ATC	TTC/CTC	Consistent
SZ48	T	T	C	T	C	GTA/GTA	ATC/ATC	CTC/CTC	Consistent
JN17	T	T	T	T	C/A	GTA/GTA	ATC/ATC	TTC/TTA	Consistent
LY55	T	T	T	T	A	GTA/GTA	ATC/ATC	TTA/TTA	Consistent
YB6	T	T	T	C/G	C	GTA/GTA	ATC/ATC	TCC/TGC	Consistent

After optimization, the final concentrations of the seven mass probes were 8.30 μmol/L (vg1016), 6.87 μmol/L (it1532), 10.12 μmol/L (fl15341), 8.91 μmol/L (fl15341-2), 9.22 μmol/L (sc1534f), 10.18 μmol/L (sc1534-2), and 7.15 μmol/L (fl15342).

### Detection limit of mPCR-MS minisequencing technology

**Fig. 3:**
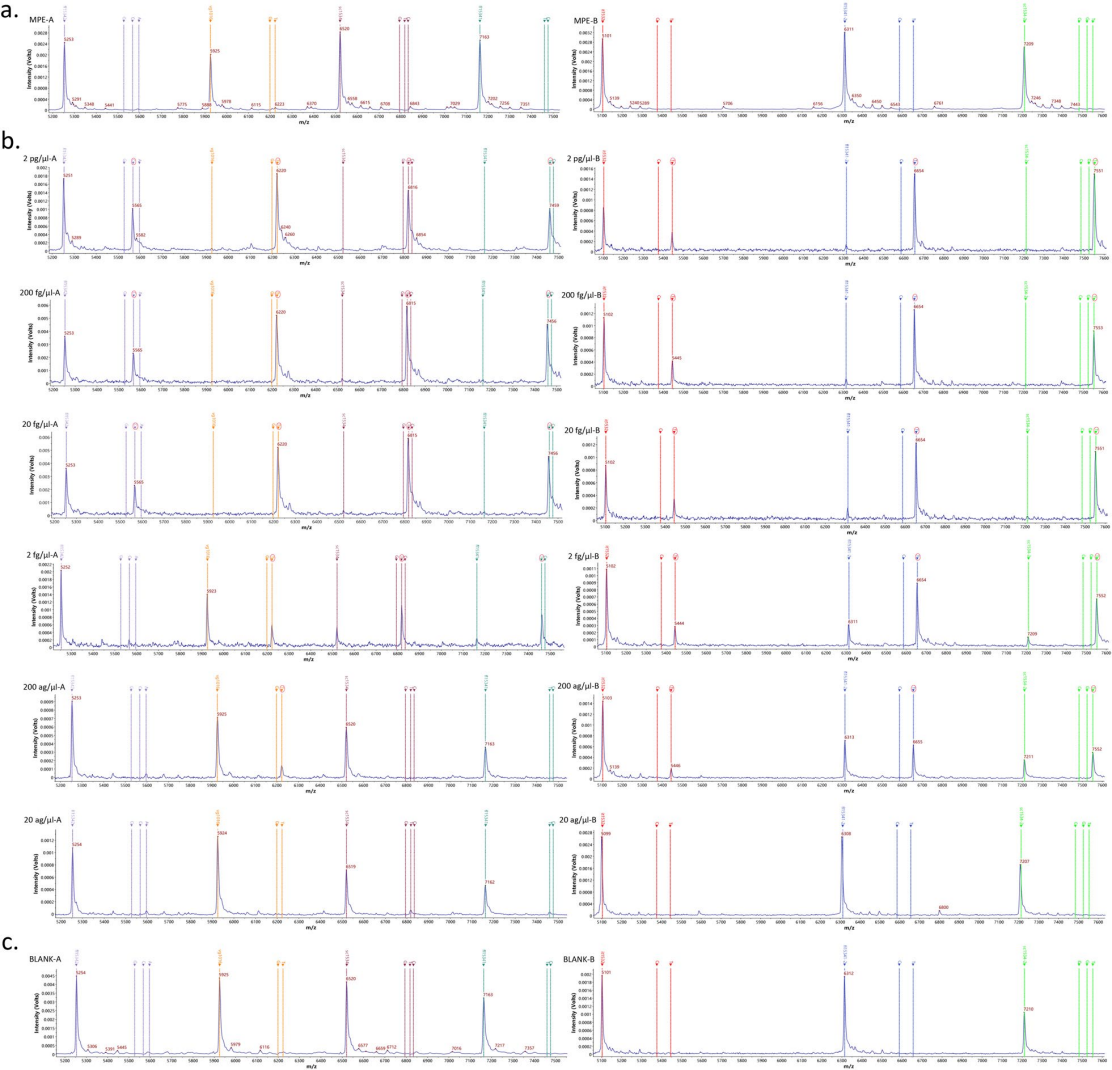
Detection limit of seven mass probes: **a** MS peaks of seven mass probes without extension; **b** the concentration of target site peaks of the mass probe extension. **c** Blank control, without DNA template target site peaks from the mass probe extension

Figure 3a–c shows the *m*/*z* of the seven mass probes without expansion, the peak of the 7 mass probes for the extension target site at different concentrations, and the 7 mass probes with expansion for the blank control, respectively. The detection limits of the seven mass probes (vg1016, it1532, fl15341, fl15341-2, sc1534, sc1534-2, fl15342) were 200 ag/μl, 200 ag/μl, 2 fg/μl, 200 ag/μl, 2 fg/μl, 200 ag/μl and 20 fg/μl, respectively. The total detection limit of all sites in MPCR-MS minisequencing technology was 20 fg/μl when using the diluted concentration of the synthetic DNA plasmids without the VGSC gene mutation (Fig. 3).

### Application of MPCR-MS minisequencing technology

The detection results for 70 samples obtained using MPCR-MS minisequencing technology are consistent with the sequencing results. The genotypes of the three VGSC gene loci in each sample can be correctly identified using our method (Table 6). An additional file shows this in more detail (see Additional file 3).

**Table 6:** Detection results of target sites in the *Aedes albopictus* VGSC gene by MPCR-MS minisequencing technology

Sample code	Base results detected by the probe					Genotype		
	vg1016	it1532	fl15341/	sc1534/	fl15342	Locus 1016	Locus 1532	Locus 1534
			fl15341-2	sc1534-2				
HNLY4, HNLY8, HNLY11, HNLY13, HNLY20, HNLY22-HNLY26, HNLY28, HNLY29, HNXC1-HNXC3, HNXC5, HNXC6, HNXC10, HNXC14, HNXC15, HNXC17, HNXC25, HNXC30, HNXC31, HNXC33, HNXC34, HNXC36, HNXC37, HNXC40	T	T	T	T	C	GTA/GTA	ATC/ATC	TTC/TTC
HNLY1, HNLY18, HNLY19, HNLY27, HNLY30, HNXC4, HNXC22, HNXC23, HNXC32, HNXC39, HNXC4, HNXC22, HNXC23, HNXC32, HNXC39	T	T	T	T/C	C	GTA/GTA	ATC/ATC	TTC/TCC
HNLY6, HNLY9, HNLY12, HNLY14-HNLY16, HNXC11, HNXC27	T	T/C	T	T	C	GTA/GTA	ATC/ACC	TTC/TTC
HNLY7	T	T/C	T	T/C	C	GTA/GTA	ATC/ACC	TTC/TCC
HNLY2, HNLY5, HNLY10, HNXC8, HNXC21	T/G	T	T	T	C	GTA/GGA	ATC/ATC	TTC/TTC
HNLY3	T/G	T	T	T/C	C	GTA/GGA	ATC/ATC	TTC/TCC
HNLY17	T/G	T/C	T	T	C	GTA/GGA	ATC/ACC	TTC/TTC
HNLY21	G	T	T	T	C	GGA/GGA	ATC/ATC	TTC/TTC
HNXC7, HNXC18, HNXC28	T	T/C	T/C	T	C	GTA/GTA	ATC/ACC	TTC/CTC
HNXC9, HNXC12, HNXC13, HNXC16, HNXC24, HNXC26, HNXC29, HNXC35, HNXC38	T	T	T/C	T	C	GTA/GTA	ATC/ATC	TTC/CTC
HNXC19	T	C	T	T	C	GTA/GTA	ACC/ACC	TTC/TTC
HNXC20	T/G	T	T/C	T	C	GTA/GGA	ATC/ATC	TTC/CTC

## Discussion

Amid the rapid emergence of new mutants of the *Ae. albopictus* VGSC gene, researchers are trying to develop fast and convenient detection methods in addition to conventional DNA sequencing. The existing technology cannot detect all mutations simultaneously. PCR coupled with first-generation sequencing technology is currently regarded as the gold standard for VGSC mutation detection in *Ae. albopictus*, which requires two PCRs to amplify the target gene followed by first-generation sequencing. Then, these identified mutation sites need to be manually examined, which is a complicated, time-consuming and high-cost process. In addition, AS-PCR is prone to contamination and has low specificity [19, 25], making it unsuitable for multiple sample detection. In contrast, the MPCR-MS minisequencing technology method that we developed in this study could detect multiple mutation sites in the VGSC gene in 96 samples within seven hours. It can simultaneously detect three mutation sites (loci 1016, 1532, and 1534) and identify the mutant genotype and its corresponding amino acid sequence. This technology has been applied to detect COVID-19 and *Mycoplasma pneumoniae* [22, 24]. *Ae. albopictus* is a multicellular eukaryotic organism. Compared with our previous studies, this study included the DNA extraction of *Ae. albopictus* rather than RNA extraction and the design of primes and probes for the target sites. DNA extraction is relatively simple compared with RNA extraction. Meanwhile, the VGSC mutation sites of *Ae. albopictus* in this study were less common than mutations studied in other microorganisms, and the design of primers and probes was less challenging.

The method that we developed here has several advantages over traditional methods. First, MPCR-MS mini-sequencing requires fewer PCR products and less sequencing than conventional PCR coupled with sequencing. The conventional PCR system is a 25 μl system [12, 21], while our newly established method is only a 10 μl system, which greatly reduces the consumption of reagents and supplies. The method established in this study greatly reduced the cost of the experiment, which is crucial in less developed countries in Asia, Africa and Latin America [26–28], where there are widespread mosquito-borne infectious diseases such as dengue fever, chikungunya and Zika [11]. The low cost and convenience render our method a great application.

This method relies on the detection of mass probes after PCR amplification, which exhibited high accuracy with no misidentification in this study. The samples that we used were one wild-type homozygotes, five wild-type and mutant heterozygotes, four homozygous mutants, and one mutant heterozygote (Table 2). We also applied this method to 70 wild-collected samples. The detection results of all samples are consistent with the sequencing results, suggesting the high accuracy of our method when testing samples. First, the MPCR-MS minisequencing technology detection results showed the base at the measured position after each probe extension, and then the genotype of each VGSC gene mutation site could be determined. Finally, the genotypes of the three VGSC gene loci in each sample could be determined. The genotypes of the three sites were correctly identified according to our judgement rules (Tables 5, 6), which were consistent with the sequencing results. The reliability of the method was verified by the detection samples, which indicated that the method has application value.

Our method also has high sensitivity. Compared with detection methods based on conventional PCR or first-generation sequencing technology, MPCR-MS mini-sequencing requires fewer PCR products while maintaining high sensitivity. We used 20 fg/μl as the LDL of this method. However, the detection limit varies among different probes. For example, the detection limit of fl15341 was 2 fg/μl after mass probe extension, while that of fl15342 was only 20 fg/μl copies. Although these two probes are in the same PCR amplification fragment, our results suggest that the probe extension efficiency of fl15342 was much lower than that of fl15341.

This method can not only detect 17 genotypes at the three sites considered in this study but also be tailored to detect other reported amino acid changes, such as TCG(S), CGC(R) and TGG(W) mutations at the 1534 locus [20]. If the probe detection results of fl15341-2, c1534-2 and fl15342 were T, C and G, then the genotype was confirmed as S/S (TCG/TCG). In addition, this method is extensible and can be used to detect new mutant genotypes at previously identified polymorphic loci. When a new mutation site is discovered, detection can be achieved by adding a corresponding probe to the new mutation site.

However, this method has some limitations. First, the 3'-terminal sequence of the extension probe is fixed, and the quality of the probe is significantly influenced by the base sequence near the detection site [22]. It is found in this study that when the probe is annealed to the template strand, the codon mismatch between the probe and the template strand will lead to inefficient detection. When a mismatch occurs, the closer it is to the detected codon, the more likely it will lead to inefficient detection. For example, when testing the locus 1534 (TCC/TCC) in the MS5 sample, the mutant is homozygous due to the mutation change of the second codon T to C. For the first codon, the probe fl15341 (reverse design) is detected, and the 3'-terminal codon of the probe is A, which mismatches the second codon C of the MS5 sample, leading to a failure of this probe to extend (Additional file 2b MS5-A). To solve this problem, the probe fl15341-2 (forward design) compensates for the lack of probe fl15341 (Additional file 2b MS5-B). Similarly, probe sc1534-2 compensates for the lack of probe sc1534. However, this conclusion is not absolute. For example, when detecting locus 1534 (CTC/CTC) of the SZ48 sample, the sc1534-2 probe was designed in the forward direction, and the 3'-terminal codon was A, which mismatches the first codon C. The second codon T was still correctly detected (Additional file 2b SZ48-B). In short, when the probe is annealed with the template strand, the codon mismatch between the probe and the template strand may lead to inefficient detection. Second, there are TCG(S), CGC(R) and TGG(W) mutations at locus 1534, which were not tested in this study due to the lack of such samples with these genotypes.

## Conclusions

MPCR-MS mini-sequencing technology is a novel and simple method for detecting VGSC gene mutations in *Ae. albopictus*. With the high accuracy, high sensitivity, time savings and economic advantages of this technology, it has great potential in mosquito surveillance, especially in Asian, African, and Latin American countries that are severely affected by insect-borne infectious diseases.

### Supplementary Information

**Additional file 1. ** The routine sequencing was used to detect VGSC genes in 70 wild-collected samples.

**Additional file 2. ** A total of 13 samples were used to establish the MPCR-MS minisequencing technology. MS peaks of the MPE probes.

**Additional file 3. ** The detection results of 70 samples using MPCR-MS minisequencing technology.

## Data Availability

The datasets used and analysed during the current study are available from the corresponding author upon reasonable request.

## Abbreviations

VGSC: Voltage-gated sodium channel
MPCR: Multiplex polymerase chain reaction
MPCR-MS minisequencing technology: Multiplex PCR-mass spectrometry minisequencing technology
MALDI–TOF MS: Matrix-assisted laser desorption ionization–time-of-flight mass spectrometry
SNP: Single nucleotide polymorphism
AS-PCR: Allele-specific PCR
*m*/*z*: Mass-to-charge ratio
SAP: Shrimp alkaline phosphatase
dNTPs: Deoxygenated nucleoside triphosphates
LDL: Lowest detection limit
3-HPA: 3-Hydroxypyridin-2-carboxylic acid
