# Supplementary material for: A novel strategy for screening mutations in the voltage-gated sodium channel gene of Aedes albopictus based on multiplex PCR-mass spectrometry minisequencing technology
Source: Infect Dis Poverty. 2023 Aug 15;12:74. doi: 10.1186/s40249-023-01122-y (PMC10426094; doi:10.1186/s40249-023-01122-y)
Supplement: Supplementary file 1 — Additional file 1. The routine sequencing was used to detect VGSC genes in 70 wild-collected samples. [file 40249_2023_1122_MOESM1_ESM.docx]

Additional file 1: The routine sequencing was used to detect VGSC genes in 70 wild-collected samples.

| Sample code | VGSC gene sequencing results | | |
| --- | --- | --- | --- |
|  | locus 1016 | locus 1532 | locus 1534 |
| HNLY4, HNLY8, HNLY11, HNLY13, HNLY20, HNLY22, HNLY23, HNLY24, HNLY25, HNLY26, HNLY28, HNLY29, HNXC1, HNXC2, HNXC3, HNXC5, HNXC6, HNXC10, HNXC14, HNXC15, HNXC17, HNXC25, HNXC30, HNXC31, HNXC33, HNXC34, HNXC36, HNXC37, HNXC40 | GTA/GTA | ATC/ATC | TTC/TTC |
| HNLY1, HNLY18, HNLY19, HNLY27, HNLY30, HNXC4, HNXC22, HNXC23, HNXC32, HNXC39 | GTA/GTA | ATC/ATC | TTC/TCC |
| HNLY6, HNLY9, HNLY12, HNLY14, HNLY15, HNLY16 | GTA/GTA | ATC/ACC | TTC/TTC |
| HNLY7, HNXC11, HNXC27 | GTA/GTA | ATC/ACC | TTC/TCC |
| HNLY2, HNLY5, HNLY10, HNXC8, HNXC21 | GTA/GGA | ATC/ATC | TTC/TTC |
| HNLY3 | GTA/GGA | ATC/ATC | TTC/TCC |
| HNLY17 | GTA/GGA | ATC/ACC | TTC/TTC |
| HNLY21 | GGA/GGA | ATC/ATC | TTC/TTC |
| HNXC7, HNXC18, HNXC28 | GTA/GTA | ATC/ACC | TTC/CTC |
| HNXC9, HNXC12, HNXC13, HNXC16, HNXC24, HNXC26, HNXC29, HNXC35, HNXC38 | GTA/GTA | ATC/ATC | TTC/CTC |
| HNXC19 | GTA/GTA | ACC/ACC | TTC/TTC |
| HNXC20 | GTA/GGA | ATC/ATC | TTC/CTC |
