# Supplementary material for: A novel strategy for screening mutations in the voltage-gated sodium channel gene of Aedes albopictus based on multiplex PCR-mass spectrometry minisequencing technology
Source: Infect Dis Poverty. 2023 Aug 15;12:74. doi: 10.1186/s40249-023-01122-y (PMC10426094; doi:10.1186/s40249-023-01122-y)
Supplement: Supplementary file 2 — Additional file 2. A total of 13 samples were used to establish the MPCR-MS minisequencing technology. MS peaks of the MPE probes. [file 40249_2023_1122_MOESM2_ESM.docx]

Additional file 2: A total of 13 samples were used to establish the MPCR-MS minisequencing technology. MS peaks of the MPE probes.

(a) MS peaks of seven MPE probes without extension.

(b) each sample target site peaks of the MPE probes extension.

(c) blank control, without DNA template target site peaks of the MPE probes extension.
