# Supplementary material for: A novel strategy for screening mutations in the voltage-gated sodium channel gene of Aedes albopictus based on multiplex PCR-mass spectrometry minisequencing technology
Source: Infect Dis Poverty. 2023 Aug 15;12:74. doi: 10.1186/s40249-023-01122-y (PMC10426094; doi:10.1186/s40249-023-01122-y)
Supplement: Supplementary file 3 — Additional file 3. The detection results of 70 samples using MPCR-MS minisequencing technology. [file 40249_2023_1122_MOESM3_ESM.docx]

Additional file 3: The detection results of 70 samples using MPCR-MS minisequencing technology.
